# Sphaeropsidin A Loaded in Liposomes to Reduce Its Cytotoxicity and Preserve Antifungal Activity Against *Candida auris*

**DOI:** 10.3390/molecules29245949

**Published:** 2024-12-17

**Authors:** Annalisa Buonanno, Maria Michela Salvatore, Antonia Feola, Antonietta Siciliano, Rosa Bellavita, Lorenzo Emiliano Imbò, Marco Guida, Anna Andolfi, Rosario Nicoletti, Angela Maione, Annarita Falanga, Emilia Galdiero

**Affiliations:** 1 Department of Biology, University of Naples Federico II, Via Cinthia, 80126 Naples, Italy; annalisa.buonanno@unina.it (A.B.); mariamichela.salvatore@unina.it (M.M.S.); antonia.feola@unina.it (A.F.); antonietta.siciliano@unina.it (A.S.); marco.guida@unina.it (M.G.); emilia.galdiero@unina.it (E.G.); 2 Department of Chemical Sciences, University of Naples Federico II, 80126 Naples, Italy; anna.andolfi@unina.it; 3 Department of Pharmacy, School of Medicine, University of Naples Federico II, Via Domenico Montesano 49, 80131 Naples, Italy; rosa.bellavita@unina.it; 4 Department of Agricultural Science, University of Naples Federico II, Via Università 100, 80055 Portici, Italy; lorenzo.emiliano.imbo@gmail.com; 5 CIRPeB, Research Centre on Bioactive Peptides “Carlo Pedone”, University of Naples Federico II, 80134 Naples, Italy; 6 BAT Center—Interuniversity Center for Studies on Bioinspired Agro-Environmental Technology, University of Naples Federico II, 80055 Portici, Italy; 7 National Biodiversity Future Center (NBFC), 90133 Palermo, Italy; 8 Council for Agricultural Research and Economics, Research Center for Olive, Fruit, and Citrus Crops, 81100 Caserta, Italy; rosario.nicoletti@crea.gov.it

**Keywords:** Sphaeropsidin A, loaded liposome, antifungal activity, biofilm, *Candida auris*, nosocomial infection, ROS production, *Caenorhabditis elegans*

## Abstract

*Candida* species constitute the most common cause of fungal infections in humans; the emergence of resistance and biofilm formation by *Candida* species further threaten the limited availability of antifungal agents. Over the past decade, *C*. *auris* has caused significant outbreaks worldwide and has emerged as a human pathogenic fungus that causes diseases ranging from superficial to life-threatening disseminated infections. Despite the recent advances in antifungal research, the mechanisms of drug resistance in *C. auris* remain poorly understood even as its ability to form biofilms poses a significant therapeutic challenge. The purpose of this research was to elucidate the fungal properties of Sphaeropsidin A (SphA), a secondary metabolite derived from *Diplodia* fungi, with a specific focus on its efficacy against *C. auris*. This study revealed that SphA and its liposomal encapsulated (SphA-L) form are fungistatic with time-kill kinetics highlighting their efficacy and significantly inhibited the formation of *C. auris* biofilms. Our investigation into the antifungal mechanism of this drug revealed notable alterations in ROS production and the disruption of the Candida cell cycle. Our findings show that SphA-L impairs key pathogenic traits of *C. auris*, such as its ability to adhere to human epithelial cell lines, while exhibiting no harmful effects on human cells, highlighting its potential as a future therapeutic agent. In *Caenorhabditis elegans* infection models, both ShpA and SphA-L displayed effective antifungal activity, significantly reducing the *C. auris* fungal load and improving nematode survival rates, underscoring their promise as antifungal candidates. Overall, the potent antifungal effects of SphA and SphA-L against *C. auris* encourage further research.

## 1. Introduction

Antimicrobial resistance (AMR) is an escalating threat to global public health, arising when microorganisms—such as bacteria, viruses, fungi, and parasites—adapt to withstand the effects of drugs, rendering standard treatments ineffective and making infections more difficult to manage. Over time, pathogenic species develop strategies to evade or neutralize antibiotics, antifungals, antivirals, and antiparasitic medications, leading to prolonged illnesses, increased healthcare costs, and higher mortality rates [1]. Biofilm populations constitute a key factor in the antimicrobial resistance observed in bacterial and fungal diseases. The National Institute of Health reports that biofilm formation is associated with 65% of microbial infections and 80% of chronic infections [2].

Antimicrobial resistance in bacterial and fungal infections is driven by several factors, including increased drug efflux through membrane transport proteins. A major contributor to this resistance, particularly in fungal diseases, is biofilm formation, which presents an ongoing challenge to public health. The most difficult mycoses to treat [3] are often caused not by planktonic cells but by sessile fungi that form structured biofilms, adhering to surfaces such as medical devices, organs, or tissues [4]. These biofilms are complex microbial communities encased in a self-produced exopolysaccharide matrix (EPM) attached to either biotic or abiotic surfaces, significantly enhancing resistance to antifungal agents [5,6,7].

Yeasts initiate biofilm formation by attaching to surfaces, proliferating, producing an extracellular matrix, and forming microcolonies. These colonies mature into biofilms from which cells disperse, colonize new surfaces, and restart the biofilm cycle. This process enhances the physical resistance of yeast cells to antifungal drugs, contributing to increased patient morbidity and mortality [8].

*Candida* species, which are typically commensal organisms on human skin and mucosal surfaces, can cause a range of infections, from superficial conditions like oral or vaginal candidiasis to severe, life-threatening systemic infections including hospital-acquired bloodstream infections [9]. The pathogenicity of *Candida* species is associated with virulence factors such as biofilm formation, yeast-to-hyphae transition, and the secretion of proteolytic and lipolytic enzymes, all linked to high mortality in hospital settings. Recently, there have been significant rises in infections caused by non-*albicans* species, including *C. auris*, an emerging nosocomial pathogen [10,11]. *Candida auris* was first described in 2009 from a patient in Japan [12]. Since its initial identification, it has caused numerous outbreaks of invasive infections in hospitals worldwide [13,14]. It is highly resistant to multiple drugs including azoles (e.g., fluconazole), polyenes (e.g., amphotericin B), and echinocandins (e.g., caspofungin) [15]. Capable of thriving in high-salt and high-temperature environments, *C. auris* forms biofilms that colonize skin and persist on medical device surfaces for up to 14 days [8], leading to invasive candidiasis with high mortality rates and causing worldwide nosocomial outbreaks, particularly in intensive care units [16], making it difficult to eradicate [17]. Biofilms of *C. auris* are thinner than those of *C. albicans* although the ability to form biofilms varies among *C. auris* isolates [8,18]. Biofilm formation allows *C. auris* to withstand harsh conditions and facilitates its spread in healthcare settings, particularly in intensive care units where central venous or urinary catheters are common [19,20]. Furthermore, several studies have indicated that the virulence of *C. auris* is comparable to, or even higher than, that of *C. albicans* [21]. Given the limited treatment options for these drug-resistant pathogens, there is an urgent need for new, effective, and safe antifungal drugs to combat *C. auris* infections [22,23,24].

**Figure 1:**
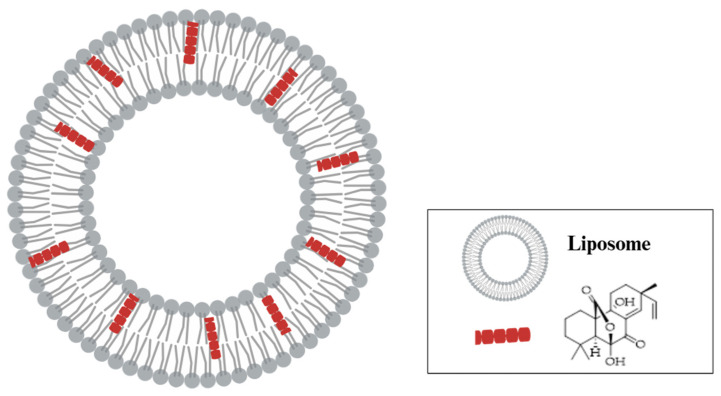
Schematic representation of liposomes loaded with SphA. The figure was created with Biorender.com (License: Academic Individual Plan × 3 yrs (27 February 2023–27 February 2026)).

There is substantial interest in developing new strategies to address and manage fungal infections by exploring new sources of bioactive compounds that offer antimicrobial activity, pharmacological effectiveness, and minimal side effects. Fungi are particularly regarded for producing compounds with a broad array of biological activities and enormous potential for exploitation in medicine, veterinary science, and agriculture [25,26]. Fungi of the genus *Diplodia* have been the subjects of intense research activities aimed at revealing the variety of low-molecular-weight metabolites with different bioactivities, including antibacterial, antifungal, and zootoxic activities [27]. Several studies have provided very important data on the interesting and promising biological activities of the pimarane diterpene Sphaeropsidin A (SphA) (Figure 1), which is a well-known product of plant pathogenic *Diplodia* species [28].

Considering the application of nanosystems for drug delivery as a strategy to improve the antimicrobial profiles of bioactive compounds and based on promising studies using SphA against bacteria, liposomes represent an interesting opportunity thanks to their ability to encapsulate molecules of different natures, both hydrophobic and hydrophilic; to their low production cost; to their biocompatibility; and, also, to their ability to reduce toxicity towards healthy cells [29]. In this perspective, we prepared liposomes loaded with SphA (SphA-L) as indicated in Figure 1.

Liposomes were characterized by dynamic light scattering (DLS) to determine the size, the zeta potential, and the stability of preparations. Moreover, the SphA loading was calculated via HPLC.

For the first time, we studied the antifungal properties of SphA, alone and encapsulated into a liposome (SphaA-L), against *C. auris,* focusing on controlling not only planktonic growth but also the inhibition of biofilm formation and mature biofilm eradication and exploring the underlying mechanisms. Furthermore, SphA and SphA-L’s cytotoxic effects on the HaCaT cell line were evaluated, as well as their ability to combat infection in vitro using an adhesion assay and in vivo using the *Caenorhabditis elegans* infection model.

## 2. Results

### 2.1. Liposome Characterization

Liposomes have achieved significant attention as versatile carriers capable of loading and delivering a wide range of molecules. The advantages of liposomal drug formulations include the protection of encapsulated drugs from chemical and biological degradation, controlled release, and reduced toxicity to healthy cells. To prepare Sph-A-loaded liposomes, DOPG/PC/Chol was used, with Sph-A added to the lipid solutions in precise amounts. Following the thin-film hydration process, unencapsulated Sph-A was removed by ultracentrifugation and quantified using HPLC. The loading efficiency was calculated by subtracting the amount of Sph-A removed from the total amount initially added. The release of SphA from liposomes was evaluated using a dialysis method. The amount of SphA released was quantified by RP-HPLC, revealing that approximately 75% of SphA was released after 24 h. Table 1 presents the encapsulation percentage for each preparation. Dynamic light scattering (DLS) analysis was conducted on both empty liposomes and SphA-L, as shown in Table 1, where all liposome formulations display a monomodal distribution with a polydispersity index (PDI) below 0.2, indicating a narrow and homogenous size distribution. All the preparations had a diameter between 108 and 121 nm. All SphA-L samples were stored at 4 °C, and their sizes were monitored over time, to obtain stability information. After 6-month storage, the sizes did not change, indicating that the liposome preparations were stable (Table 1).

The analysis of the zeta-potential did not show a change between empty liposomes and those loaded with the maximum amount of SphA, which indicated no changes in the surfaces of the liposomes upon SphA encapsulation (Table 2). Indeed, the hydrophobic nature of the loaded compound allowed the interaction with hydrophobic tails (Figure 1).

### 2.2. Anti-Candida Activity of SphA and SphA-L Against Planktonic and Sessile Growth

We evaluated the fungicidal effects of SphA and SphA-L on *C. auris* by determining the MIC (minimum inhibitory concentration) and MFC (minimum fungicidal concentration) values. The results showed MIC values of 35 μg mL^−1^ for SphA and 50 μg mL^−1^ for SphA-L, and MFC values more than 100 μg mL^−1^ for both, indicating fungistatic activity (Table 3).

**Figure 2:**
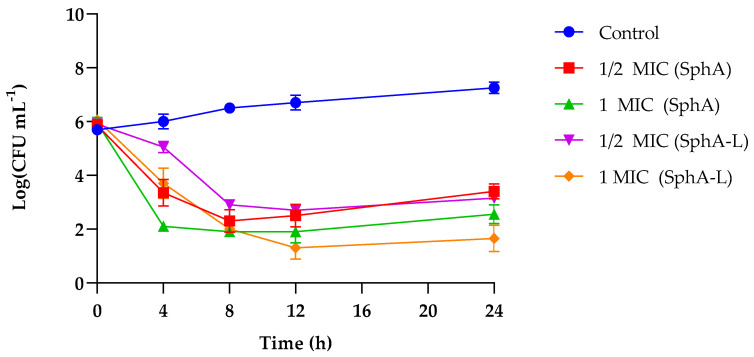
Time to kill assay of SphA and SphA-L on *C. auris* at concentrations of 17.5 μg mL^−1^ (1/2 MIC) and 35 μg mL^−1^ (1 MIC) of SphA and 25 μg mL^−1^ (1/2 MIC) and 50 μg mL^−1^ (1 MIC) of SphA-L. Data reported are the means of three independent experiments ± SDs.

The time- and concentration-dependent activity of the formulations made SphA applicable as an antimicrobial drug. Here, we investigated the antifungal effects of SphA and SphA-L on *C. auris* at different time intervals and at two different concentrations. The results showed that the antifungal effect of SphA peaked at the 8th hour, decreasing the microbial concentration of 3.9 Log at a concentration of ½ MIC and of 4.3 Log at a concentration of 1 MIC (Figure 2). However, over time, this effect diminished, remaining constant for 24 h. Furthermore, when the isolates were grown with the two different concentrations of SphA-L, a much slower but clear and strong effect was noticed at 12 h, exhibiting a decrease of about 5 Log for both concentrations until 24 h.

### 2.3. Antibiofilm Activity of SphA and SphA-L Against C. auris

**Figure 3:**
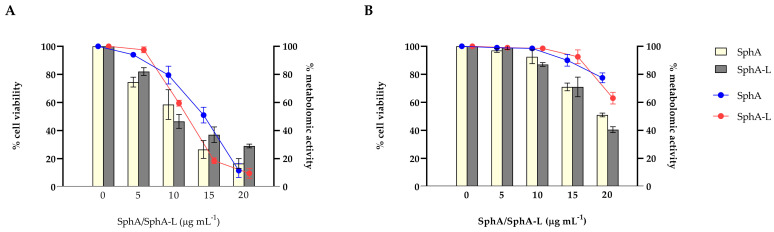
Efficacy of SphA and SphA-L on *C. auris* at different stages of biofilm development. The sensitivity of *C. auris* biofilm was reported as viable cell number (%) (bar graph) and metabolic activity (line graph). SphA and SphA-L were added to attached cells and biofilms monitored following a 24 h period (**A**) or added to cells in a 24 h biofilm and monitored following an additional 24 h growth period (**B**). Data from CFU and XTT assays are represented as percent differences relative to untreated biofilm cells. Data reported are the means of three independent experiments ± SDs.

We also assessed the efficacy of SphA and SphA-L towards early and mature *C. auris* biofilms. SphA exhibited excellent inhibitory effects against the biofilm formation and a trend of dose-dependent reduction in relative metabolic activity and the viable cell number was observed to prevent *C. auris* biofilm formation (Figure 3A). The sub-MIC concentration of 20 µg mL^−1^ decreased both total biofilm biomass and vital biomass; otherwise, the lowest concentration tested had no significant impact on the biofilm formation. The same result was observed for SphA-L: the highest concentration tested showed a 75% reduction in the total biofilm biomass with a reduced metabolic activity, even if at a lower extent than SphA.

Next, we measured the impacts of SphA and SphA-L on preformed biofilms (Figure 3B). After 24 h of development, biofilms were treated with different sub-MIC concentrations ranging from 0 to 20 μg mL^−1^, the impacts of which were measured after an additional 24 h. Mature biofilm seemed to be more tolerant. The higher concentration of SphA tested was required to eliminate about 60% of total biofilm biomass, showing a moderate eradication ability. The same concentration of SphA-L caused 50% of eradication, indicative of a similar sensitivity profile.

### 2.4. Flow Cytometry Cell Cycle Analysis of C. auris Cells Treated with SphA and SphA-L

**Figure 4:**
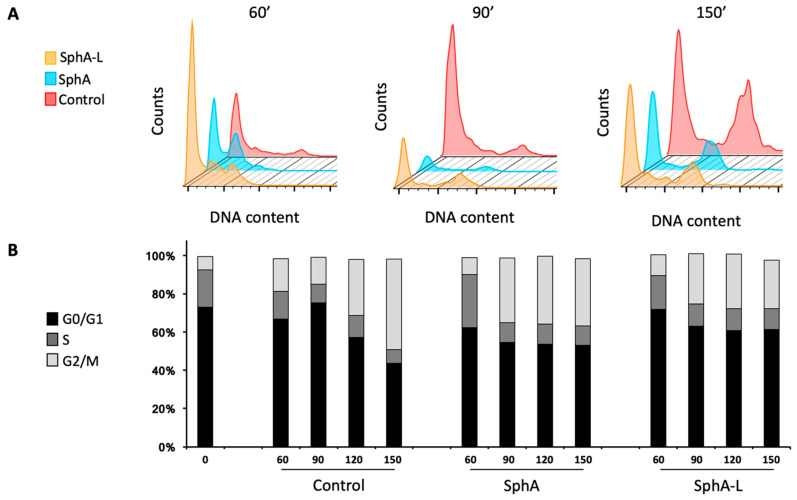
FACS flow cytometry analysis of *C. auris* by using propidium iodide staining (PI). (**A**) Flow cytometry analysis showed the DNA contents at the indicated time points of *C. auris* treated or not with SphA and SphA-L. Cell cycle analysis was obtained with the BD Accury C6 flow cytometer. The data were analyzed with the 10.6 version of FlowJo Program. (**B**) Histogram represents the mean percentages of at least three independent experiments in each cell cycle phase of untreated control and SphA and SphA-L treated cells at 0, 60, 90, 120, and 150 min.

To corroborate these data, we evaluated whether SphA and SphA-L had an impact on the *C. auris* cell cycle through flow cytometry analysis. To achieve this aim, we synchronized >90% (97.4%) of cells at the G0/G1 phase as previously described by S K Patel et al. [30] to avoid a chemical reagent that could affect the native cellule physiology (Figure S3). Then, we allowed growth until 150 min in the presence or not of SphA and SphA-L 10 μg mL^−1^, collecting 10^5^ cells from each experimental point every 30 min. As shown in Figure 4, accordingly with the number of genome copies present in single cells, starting at the left, the first peak represented the number of cells in the G1 phase; the lag phase corresponded to cells in the S phase; the last peak corresponded to the G2/M-phase. Our results reported a cell cycle analysis of *C. auris* that showed a progression of about 15% in the S phase after 60’ whereas it was 48% in the G2/M phase within 150’. The about-25% of *C. auris* cells treated with 10 μg mL^−1^ of SphA were in the S phase for only 60 min. Only 33% of the cells went into the G2/M phase at 90 min and, despite time going on, we did not observe an increase in the number of cells in the G2/M phase compared to what was noted after 90 min of the treatment.

It is noteworthy that the global count in each cell cycle phase was less than the untreated control, compatible with a cytotoxic effect. Although no differences were seen between the two formulations, we can say that the best effect was from SphA alone.

### 2.5. Effects of Reactive Oxygen Species (ROS) Production in SphA and SphA-L Treated Yeast Cells

**Figure 5:**
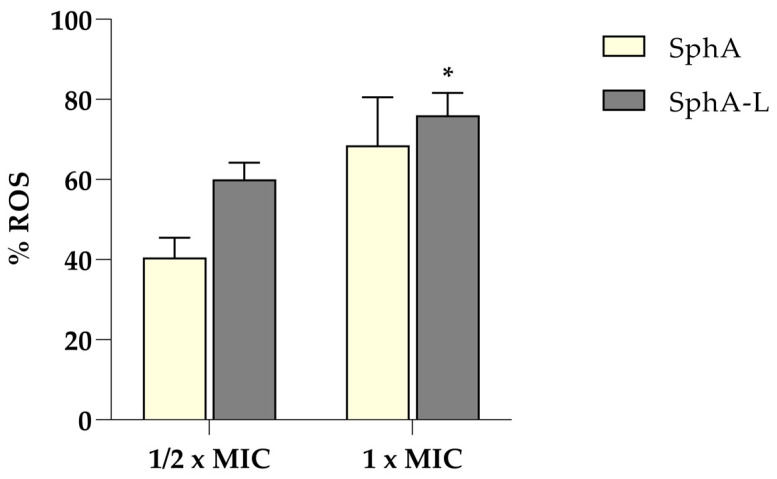
Susceptibility of *C. auris* to SphA and SphA-L induced intracellular ROS production. ROS production was assessed by cell staining with H_2_DCFDA. Data represents the means ± SDs of three independent experiments; statistical significant is indicated by * *p* < 0.05 (Tukey’s test).

The buildup of reactive oxygen species (ROS) within cells can lead to DNA and RNA damage, increasing oxidative stress that disrupts essential cellular components and results in cell death. To assess whether the inhibitory effects of SphA and SphA-L were associated with ROS generation, ROS levels were evaluated using H_2_DCFDA, which penetrates the cell membrane and reacts with ROS. Figure 5 shows the significantly increased green fluorescence in treated cells in both the ½ MIC and 1 MIC groups compared to the untreated cells that showed no detectable green fluorescence. These results suggest that both SphA and SphA-L induce ROS and elevate oxidative stress; consequently, we hypothesize that the induced excess ROS generation influenced the antifungal effect.

### 2.6. Cell Cytotoxicity Assay

**Figure 6:**
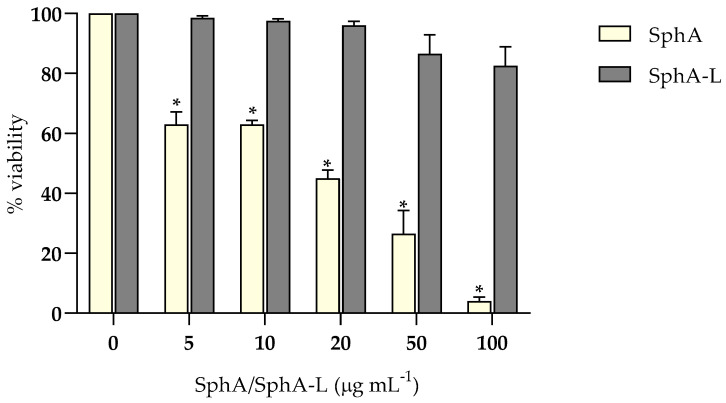
Cytotoxic effects of SphA and SphA-L on HaCaT cells, measured by MTT assay. Various concentrations of SphA and SphA-L (5 μg mL^−1^, 10 μg mL^−1^, 20 μg mL^−1^, 50 μg mL^−1^, and 100 μg mL^−1^) showed dose-dependent responses after a 24 h incubation period. Cell viability was measured at OD_595_ with means ± SDs (n = 3), and statistical significance is indicated by * *p* < 0.05, denoting significance when compared to the untreated cells (Tukey’s test).

The cytotoxic effects of SphA and SphA-L on HaCaT cells were profiled using the MTT assay at concentrations ranging from 5 to 100 μg mL^−1^. In particular, as reported in Figure 6, a statistically significant (*p* < 0.05) decrease in cell viability was evident for SphA already at a low concentration (5 μg mL^−1^), showing 40% of cytotoxicity; this effect increased up to 90% at the highest concentration tested. On the contrary, SphA-L showed a slight cytotoxicity (less than 10%) only at the highest concentrations tested. Liposomal encapsulation can have a significant impact on a drug’s functional properties compared to its unencapsulated counterpart. The chemical composition and physical form of the liposomes can also influence the functional characteristics of the drug.

Hence, the subsequent experiment of adhesion was conducted only with SphA-L.

### 2.7. Adhesion to HaCaT Cells

**Figure 7:**
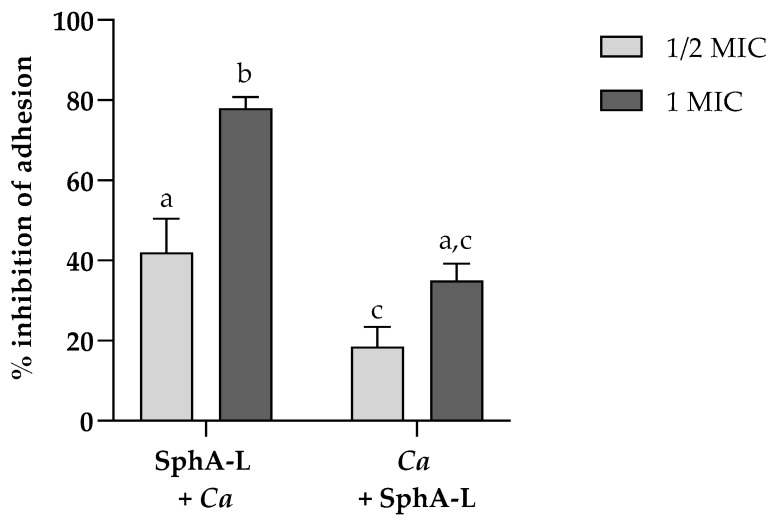
Evaluation of the anti-adhesion effect of SphA-L on *C. auris* cells using HaCaT cells that were either pre-treated or post-treated. *C. auris* infection of HaCaT cells without treatment served as the positive control. Results represent the means of three independent experiments, with error bars indicating standard deviation. Values with dissimilar letters were significantly different from each other (a, b, c) (*p* < 0.05, Tukey’s test).

To evaluate whether SphA-L could reduce or inhibit the adhesion of *C. auris* on HaCaT cells, SphA-L at concentrations of 25 and 50 μg mL^−1^ was added to cells before challenging with the yeast or after the infection (Figure 7). A decrease in adhesion to HaCaT cells was observed when cells were pre-treated with the compound. These data clearly indicated that it was possible to protect the epithelial cells from this yeast pathogen invasion and that the cell pre-treatment had a better inhibitory effect.

### 2.8. In Vivo Effect of SphA and SphA-L in C. elegans

**Figure 8:**
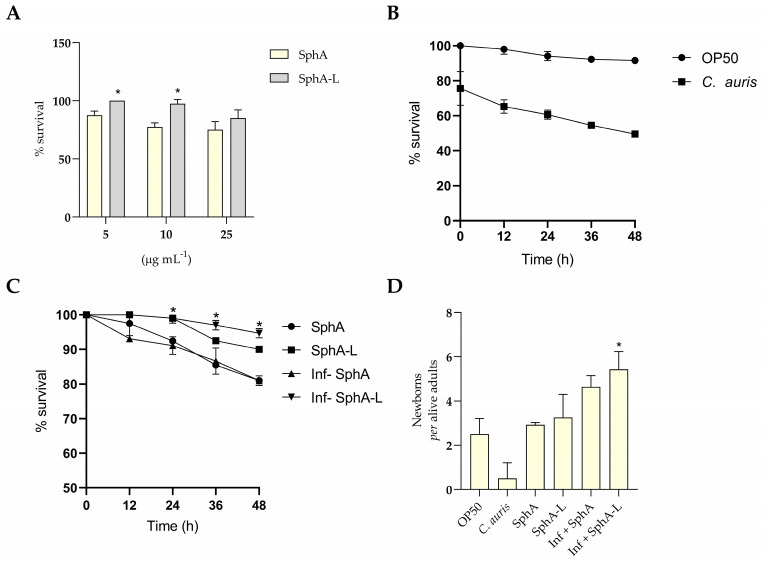
In vivo evaluation of the antifungal efficacy of SphA and SphA-L in *C. elegans*. (**A**) Pilot screen of *C. elegans* survival in the presence of SphA and SphA-L. The liquid assay assessed the survival of infected and non-infected nematodes over a 48 h period. Asterisks (*) indicate statistically significant differences between treatments, *p* < 0.05. (**B**) Survival rates of *C. elegans* infected with *C. auris*. This figure illustrates the comparative survival of nematodes exposed to *E. coli* OP50 as a negative control and those infected with *C. auris*. The survival rates were statistically different, *p* < 0.05. (**C**) Antifungal effects of SphA and SphA-L on *C. elegans*. The survival of infected and non-infected nematodes was evaluated in the presence of SphA and SphA-L over 48 h. Asterisks (*) indicate statistically significant differences of SphA-L compared the other treatments, *p* < 0.05. (**D**) Average number of offspring produced per nematode in various treatment groups. This figure presents the reproductive outputs of both infected and non-infected nematodes treated with SphA and SphA-L as well as controls. Asterisks (*) indicate statistically significant differences between treatments and the control group (OP50), *p* < 0.05.

The in vivo efficacy of SphA and SphA-L as antifungals against *C. auris* was evaluated in a widely accepted nematode model involving *C. elegans*. A pilot screen was conducted in a 24-well plate format using a *C. elegans* liquid assay to evaluate SphA and SphA-L, which allowed for the survival of infected worms (Figure 8A). The toxicity of both SphA and SphA-L was tested on *C. elegans* at sub-inhibitory concentrations ranging from 5 μg mL^−1^ to 25 μg mL^−1^. No significant differences in survival were observed between worms treated with SphA (75%) and those treated with SphA-L (85%) after 48 h, indicating that both formulations were well tolerated within this concentration range.

Subsequently, the survival of *C. elegans* was monitored using *E. coli* OP50 as a negative control and *C. auris* as a positive control for infection (Figure 8B). The nematodes were infected with *C. auris* while those exposed to *E. coli* OP50 served as a baseline for normal survival. Over a 48 h period, the survival of *C. elegans* remained high in the OP50-treated group (91%) whereas in the *C. auris*-infected group, survival progressively declined, reaching 50% at 48 h.

To determine the antifungal effect, *C. elegans* specimens, infected and non-infected, were treated in the presence and absence of SphA and SphA-L at a concentration of 10 μg mL^−1^ (Figure 8C). The survival of both infected and non-infected *C. elegans* treated with SphA and SphA-L was evaluated over a 48 h period. For non-infected nematodes, the survival rates remained relatively stable, with 80% for SphA and 90% for SphA-L at 48 h, indicating good tolerance to both compounds.

In contrast, the survival of *C. auris*-infected nematodes treated with SphA demonstrated a marked decline over time while those nematodes treated with SphA-L exhibited high survival rates. At 48 h, the survival rate for infected nematodes treated with SphA was 82% whereas those treated with SphA-L showed a significantly higher survival rate (95.7%). These results highlight that SphA-L was more effective than SphA in enhancing the survival of *C. elegans* infected with *C. auris*, suggesting its potential in superior antifungal treatment.

In addition to the survival analysis, the number of offspring produced per nematode was counted across the different treatment groups (Figure 8D). The average number of neonates per nematode was 2.5 for *E. coli* OP50 while *C. auris* resulted in a significantly lower average of 0.5. Nematodes treated with SphA produced an average of 2.7 neonates and those treated with SphA-L had an average of 3.4. Infected nematodes treated with SphA exhibited an average of 4.29 neonates while those treated with SphA-L showed the highest average at 4.88.

These results indicated that nematodes infected with *C. auris* produced significantly fewer offspring compared to those treated with SphA and SphA-L. Furthermore, the highest average number of neonates was observed in infected nematodes treated with SphA-L, suggesting that this compound may enhance reproductive fitness even in the presence of an infection.

## 3. Discussion

Because an excessive or inappropriate use of antibiotics in human medicine, agriculture, and livestock can accelerate the development of resistance, without urgent action, it has been predicted that drug-resistant infections could cause millions of deaths worldwide annually by the middle of the century. The high rate and spread of *Candida* infections in the community represent an important issue that contributes to resistance to common antifungal agents. A critical and unmet challenge remains the urgent need for the development of novel classes of antifungal drugs. Natural bioactive compounds offer a promising strategy for addressing this issue. *Diplodia corticola*, collected from a natural site in Sardinia, has been identified as an efficient producer of diverse secondary metabolites, including Sphaeropsidins A and C, sapinofuranones C and D, diplopyrone B, diplopyrone, diplobifuranylones A-C, diplofuranone A, and the (S,S)-enantiomer of sapinofuranone B. Using a deep learning approach, Evidente et al. discovered that SphA exhibits broad-spectrum bactericidal, insecticidal, herbicidal, and potent anticancer activities, operating through a unique mechanism of action. While SphA demonstrated moderate in vitro cytotoxicity, it showed IC50 values ranging from 6.74 to 46.64 µM against the KB, SGC-7901, SW1116, and A549 cell lines [28,29,30]. Roscetto et al. [31] stated that Sphaeropsidin A not only appeared to have antimicrobial potential towards some Gram-positive and Gram-negative bacteria but also was capable of reducing biofilm formation. In addition, the same authors reported a cytotoxic activity on HaCat cells that affected cell viability at about 40–45%, even at low concentrations.

Natural plant-based compounds often encounter stability challenges due to their volatility and susceptibility to photolysis and oxidation. Additionally, their low solubility can limit their antimicrobial effectiveness. Employing microencapsulation techniques can enhance the stability and efficiency of these compounds. The global rise in *C. auris* infections and its resistance to existing drugs have heightened concerns, emphasizing the urgent need to develop new therapeutic options [32].

The objective of this study was to evaluate the action of SphA alone and loaded in liposomes against the planktonic and sessile (biofilm) cells of *C. auris* in vitro and in vivo with reference to the *C. elegans* infection model and cytotoxicity on the HaCaT cell line.

Since Bangham’s initial discovery of liposomes in 1963, the concept of using these small spherical vesicles for drug encapsulation has gained significant traction [33]. Liposomes offer the unique ability to encapsulate substances and release them in a controlled manner over time, profoundly influencing a drug’s functional properties, including its chemical composition and physical form.

Homogenous and stable liposomes showed a diameter of about 120 nm and a zeta potential that did not change in the presence of SphA, indicating its interaction with the hydrophobic tails. The SphA encapsulation of each liposomal formulation was quantified via HPLC and the biological activity was tested.

Low MIC values indicate a higher practical potential for an active compound. In this study, SphA and SphA-L demonstrated effectiveness against *C. auris* in planktonic cultures, with MIC values of 35 and 50 μg/mL, respectively. The antimicrobial nature of a substance against a particular microorganism is classified based on the MFC/MIC ratio: an MFC/MIC ratio ≤2:1 indicates a fungicidal effect while a ratio >2:1 suggests a fungistatic effect [34]. Our results showed a fungistatic action against *C. auris* from both formulations. The fungistatic action of the compounds was confirmed by the time to kill, which highlighted, with respect to time and concentration, the fungistatic behavior of the two formulations after 8 and 12 h, respectively, emphasizing a slower effectiveness when SphA was encapsulated in the liposome. The action of SphA and SphA-L on biofilms of *C. auris* caused an almost complete reduction in biofilm at the highest sub-MIC concentration tested for SphA while for the encapsulated compound, the same concentration was necessary to obtain 70% of inhibition; the metabolic activity of cells within biofilms was higher as compared to that of biofilms treated with SphA alone. The mature biofilm was more resistant and the eradication capacity of the two compounds was lower at all the concentrations tested.

Moreover, our results agreed with several previous studies reporting cell cycle arrest in the G2/M phase when *Candida* cells were treated with different external agents [35,36]. In our experiments, both compounds induced the cell cycle’s S phase already after 60 min, arresting the cells in the G2/M phase between 90 and 150 min and not allowing the passage into mitosis. In any case, the impairment of the cell cycle triggered the fungal cell morphological changes that increase the recognition of *Candida* cells by the host immune system. Both substances led to increased ROS production in the treated *C. auris* cells, thereby placing cells under oxidative stress, emphasizing that their mechanism of action, at least in part, was due to ROS formation. Fortunately, no cytotoxicity of SphA-L was observed in HaCaT cells compared to the non-encapsulated substance, which was significantly cytotoxic even at low concentrations. SphA-L shows low affinity for HaCaT cells, likely due to the presence of anionic lipids (DOPG). These negatively charged lipids reduce interactions with HaCaT cells, which also possess a negative surface charge [37]. Conversely, the same anionic lipids enhance interactions with positively charged components on fungal membranes while enhancing interactions with positively charged elements on fungal membranes [38].

Consequently, the evaluation of the ability to inhibit the adhesion of *C. auris* to HaCaT cells by pre-treatment or post-treatment only with SphA-L highlighted that the pre-treatment was more effective at both the concentrations tested.

*C. elegans* are transparent, non-parasitic worms with a simple anatomy and short life cycle, making them ideal for studying neurobiological behavior, gene expression, and the toxic effects of potential drugs. The final part of this study evaluated the in vivo antifungal efficacy of SphA and SphA-L using a *C. elegans* model infected with *C. auris.* The results showed that SphA-L exhibited superior antifungal activity compared to SphA, significantly reducing the fungal burden in *C. elegans.* Furthermore, SphA-L was less toxic, demonstrating not only greater survival in worms infected and treated with SphA-L but also their greater reproductive capacity during the observation time.

All these qualities provide an additional advantage for studying the potential use of this drug for the treatment of infected patients.

## 4. Materials and Methods

### 4.1. Materials

Dioleoyl-sn glycero-3-phospho-(10-rac-glycerol) sodium salt (DOPG), cholesterol (Chol), phosphatidylcholine (PC), 2,7 dichlorodihydrofluorescein diacetate (H_2_DCFDA), dimethylsulphoxide (DMSO), Acido 3-(N-morfolino) propan solfonico, Acido 4-morfolinpropansolfonico (MOPS), 2,3-bis(2-methoxy-4-nitro-5-sulfo-phenyl)-5-[(phenylamino)carbonyl]-2H-tetrazolium hydroxide (XTT), 3-(4,5-Dimethyl-2-thiazolyl)-2,5-diphenyl-2H-tetrazolium Bromide, Thiazole Blue (MTT), trypsin/EDTA solution, and l-glutamine, and 1% penicillin/streptomycin were purchased from Sigma-Aldrich/Merck.

### 4.2. Production, Isolation and Identification of Sphaeropsidin A

Sphaeropsidin A was extracted from the fungus *Diplodia corticola* (MAEC10) recovered from cork oak (*Quercus suber*) exhibiting dieback symptoms and cankers in Algeria. A liquid culture of *D. corticola* was prepared as previously reported [39]. Briefly, mycelial plugs from actively growing cultures were inoculated in Czapek-Dox broth (Oxoid, Thermo Scientific, Waltham, MA, USA) amended with 2% cornmeal in 500 mL Erlenmeyer flasks containing 250 mL of the substrate. The culture was incubated in darkness on a stationary phase at 25 °C for 30 days. After the incubation period, the liquid phase was separated by filtration through Whatman No. 5 filter paper. The culture filtrate was extracted three times with ethyl acetate (EtOAc) at native pH (=6). The organic phase was dried with anhydrous Na_2_SO_4_ and evaporated under reduced pressure, yielding crude extract as brownish solid residues. The crude extract was submitted to fractionation by column chromatography and thin-layer chromatography on silica gel eluted with diverse polarity solvents.

Sphaeropsidin A (white solid) was identified by HPLC-ESI-HRMS and by comparing NMR data with previously reported data) [39]. ^1^H NMR spectrum (Figure S1) was recorded on a Bruker AMX instrument at 400 MHz in CDCl_3_. The same solvents were used as internal standard. HRESI-TOF mass spectrum (Figure S2) was measured on an Agilent Technologies ESI-TOF 6230DA instrument (Milan, Italy) in the positive ion mode.

### 4.3. Liposome Preparation and Characterization of Chemical Physical Properties

Liposomes were prepared by the thin lipid film hydration procedure. Mixed solutions of DOPG/PC/Chol (24:61:15 weight ratio) were prepared by dissolving the lipids (total lipid amount 1.5 mg mL^−1^) in chloroform in presence of desired amount of SphA dissolved in ethanol. In particular, we prepared four different solutions with the following amounts of SphA: 20 μg mL^−1^, 50 μg mL^−1^, 100 μg mL^−1^, and 200 μg mL^−1^ and empty liposomes. The prepared solutions were combined, and the solvent was evaporated by gently rotating the solution-containing tube under a nitrogen stream, followed by overnight lyophilization. The resulting films were then resuspended in phosphate-buffered saline (PBS) at pH 7.4 and vortexed for 1 h. Subsequently, the lipid suspensions were passed through a polycarbonate membrane with a 100 nm pore size using a thermobarrel extruder, repeating the extrusion process ten times (Northern Lipids Inc., Burnaby, BC, Canada). Unloaded SphA was removed through ultracentrifugation using a Beckman Coulter, Opima^TM^ MAX-XP Ultracentrifuge (rotor TLA 100.3), for 3.5 h, at 4 °C, 40,000 rpm.

Entrapment efficiency was assessed via reversed-phase high-performance liquid chromatography (RP-HPLC) using a gradient of MeCN (0.1% TFA) in H_2_O (0.1% TFA) ranging from 30% to 60% over 30 min. The encapsulated SphA content was quantified by subtracting the amount of unencapsulated SphA from the total loaded amount.

The hydrodynamic diameters (DHs) and polydispersity index (PDI) of SphA-L and empty liposomes were measured by dynamic light scattering (DLS) using a Malvern Zetasizer Nano ZS (Malvern, UK). Zeta potential measurements for both SphA-L and empty liposomes were also conducted with the Malvern Nano ZS. The analyses used a 4 mW He-Ne laser at 633 nm with a scattering angle fixed at 173° and a temperature of 25 °C. Each sample was measured in triplicate and each measurement was repeated three times.

### 4.4. In Vitro SphA Release from Liposomes

The in vitro release of SphA from SphA-L was assessed using a dialysis method. In brief, SphA-L (with any free SphA removed) was placed in a dialysis bag (with a molecular weight cutoff of 1000 Da) and dialyzed against PBS while being continuously stirred at 37 °C. At 24 h, the concentration of SphA released was determined by RP-HPLC using a previously established calibration curve.

### 4.5. Microorganisms and Cells

In this study, *C. auris* strain DSM 21092 was used and cultured under different experimental conditions. Fungi from freezer stocks were streaked on YPD (Becton, Dickinson, and Company) agar plates (1% yeast extract, 2% peptone, 2% dextrose, and 2% agar) and incubated at 30 °C for at least 20 h; then, single colonies were selected and grown in YPD medium (1% yeast extract, 2% peptone, and 2% dextrose) prior to each experiment of MIC or MFC. Otherwise, for adherence and biofilm formation, *C. auris* was cultured in RPMI-1640 medium (Thermo Fisher Scientific, Waltham, MA, USA). The HaCAT cell line (non-tumorigenic human keratinocyte cells) was purchased from ATCC (American Type Culture Collection, Manassas, VA, USA). The cells were maintained in Dulbecco’s Modified Eagle Medium (DMEM, Sigma-Aldrich, St. Louis, MO, USA) supplemented with 10% Fetal Bovine Serum, 1% l-glutamine, and 1% penicillin/streptomycin (Sigma-Aldrich) in a humidified incubator at 37 °C and 5% CO_2_. Once 70–80% confluency was reached, the cells were detached with trypsin/EDTA solution (Sigma-Aldrich) and cultured into new flasks.

### 4.6. Anti-Candida Activity of SphA and SphA-L Against Planktonic Growth

The antifungal activity of the SphA and SphA-L was determined in 96-well microtiter plates using the broth microdilution protocol of the Clinical and Laboratory Standards Institute (CLSI) document M27-A3 [40,41]. Briefly, 90 μL logarithmic-phase *C. auris* cells (1.0 × 10^5^ CFU mL^−1^) were added into a 96-well microplate preloaded with 10 μL of the two compounds at final concentrations ranging from 0 to 100 μg mL^−1^. The plates were incubated at 30 °C for 24 h. The MIC was defined as the lowest compound concentration that completely inhibited fungal growth. For minimum fungicidal concentration (MFC), 50 μL from each no turbid well identified during MIC determination was spread on YPD agar plates and incubated for 24 h at 30 °C. The MFC was defined as the lowest compound concentration at which no colony was observed on plates. Since the MFC of both was indeterminate up to 100 μg mL^−1^, the subsequent experiments were performed with SphA and SphA-L at 35 and 50 μg mL^−1^ concentrations (½ MIC and MIC), respectively.

### 4.7. Kinetics of Yeast Killing

The kinetics of *C auris* killing were investigated by inoculating about 1 × 10^6^ CFU mL^−1^ of yeast in YPD medium containing the compound at concentration of ½ MIC to 1 MIC as previously described [42]. Aliquots of 10 μL were withdrawn at selected time points and serially diluted in PBS to stop further reaction before plating on YPD agar until 24 h. CFUs were calculated based on the average of triplicate plates. The results were shown in Log_10_ (CFU mL^−1^).

### 4.8. Biofilm Formation and Treatment Activity

The antibiofilm activity of antifungals was evaluated during biofilm formation and on pre-formed biofilms of *C. auris.* For biofilm formation, 100 μL of 1 × 10^6^ CFU mL^−1^ of *C. auris* in RPMI 1640 medium buffered with 0.16 M MOPS (RPMI-MOPS) was dispensed in 96-well flat microplates and incubated for 1.5 h at 37 °C with shaking (150 rpm) for the adhesion phase. Then, the supernatant was removed, wells were washed twice with PBS, and 100 μL of medium was added to each well for biofilm formation by incubation at 37 °C with shaking (150 rpm) for 24 h. To evaluate the antifungal activity on biofilm formation, 100 μL of RPMI-MOPS medium containing different sub-MIC concentrations of SphA and SphA-L was added to each well after the adhesion phase; the plates were incubated until 24 h at 37 °C with shaking and the detection of vital biomass was conducted. In order to evaluate the effect of antifungals on pre-formed biofilms, supernatants (containing dispersion cells) were removed from each well after 24 h of incubation and the sessile cells were treated with SphA and SphA-L for additional 24 h at 37 °C. After treatments, the metabolic activity of biofilm cells was quantified using the 2,3-bis(2-methoxy-4-nitro-5-sulfo-phenyl)-5-[(phenylamino)carbonyl]-2H-tetrazolium hydroxide (XTT) reduction assay [43]. The antifungal concentration that caused ≥50% reduction in the metabolic activity of the biofilm compared with the control (incubated in the absence of the antifungal agent) was determined and the lowest concentration resulting in ≥50% reduction in the metabolic activity was considered as the biofilm MBIC or MBEC [44]. For CFU count, biofilm structure was disrupted, and cells were serially diluted in PBS and spread on YPD plates. After 24 h growth at 37 °C, colonies were counted and data were reported as the relative percent cell viability values compared to untreated biofilms.

### 4.9. Effect of Reactive Oxygen Species (ROS) Production in SphA and SphA-L Treated Yeast Cells

The quantification of ROS accumulated in *C. auris* was performed using the H_2_DCFDA assay to determine whether the inhibitory effect of SphA and SphA-L was linked to ROS induction [42]. After treatments (½ MIC and 1 MIC) for 4 h, yeast cells were stained with a final concentration of 10 μM H_2_DCFDA and incubated in the dark at room temperature for 30 min. The stained cells were centrifuged at 1500× *g* for 10 min and washed with PBS to eliminate excess dye. The fluorescence intensities of resuspended cells were determined with the Varioskan LUX (Thermo Scientific) microplate reader. The excitation and emission wavelengths for green fluorescence were 488 and 535 nm, respectively.

### 4.10. Flow Cytometry Cell Cycle Analysis of C. auris Cells Treated with SphA and L-SphA 20 μg mL^−1^

*Candida auris* cells were synchronized as previously described [30], then allowed to grow for 150 min in the presence and in the absence of 10 μg mL^−1^ SphA and SphA-L and harvested every 30 min at a concentration of 1 × 10^5^ to monitor cell cycle progression. Cells were washed, fixed in 70% ethanol for 12 h, rehydrated for 5 min with 1mL of Tris MgCl^2+^ buffer (10 mM Tris, 5 mM MgCl^2+^), and treated with 0.5% pepsin for 1 h at room temperature. Subsequently, the cells were washed twice with Tris MgCl^2+^ buffer and supplemented with 25 μL of 10 mg mL^−1^ RNAse A at 30 °C. After 3 h, 30 μL of proteinase K (20 mg mL^−1^) was directly added into the samples at 50 °C overnight. The day after this, the cells were stained with 500 μL of 50 μg mL^−1^ of propidium iodide for 75 min at room temperature. Cell cycle analyses were obtained with the BD Accury C6 flow cytometer. The data were analyzed with the 10.6 version of FlowJo Program.

### 4.11. Cell Cytotoxicity Assay

The cytotoxic effects of the SphA and SphA-L on HaCat cells were assessed using MTT assay. In brief, the cells were cultivated in RPMI medium supplemented with 10% FBS (*v*/*v*) and 1% penicillin–streptomycin cocktail (*v*/*v*) in presence of 5% CO_2_ at 37 °C in a humidified incubator. After reaching a confluency of more than 80%, the cells were harvested by trypsinization. Subsequently, 1 × 10^4^ cells mL^−1^ (100 μL) were seeded in 96-well culture plates and incubated for further 24 h under the same conditions. Following incubation, the culture medium was replaced with 100 μL of fresh medium containing different concentrations of the test compounds (range: 5–100 μg mL^−1^). Cells viability was determined by MTT assay. Briefly, 10 μL of MTT solution (5 mg mL^−1^) was added to each well after treatments to reach a final concentration of 0.5 mg mL^−1^. As a result of 4 h incubation in a humidified incubator at 37 °C and 5% CO_2_, the formazan crystals formed were dissolved with DMSO. The absorbance of formazan crystal concentrations was read at 570 nm in a microplate reader. The cell viability (%) was calculated as follows: (OD of treated cells/OD of control) × 100. As internal control, cells containing only dimethylsulphoxide (DMSO) (Sigma-Aldrich, D8418-100 mL) as the solvent at the same concentration used in each test wells were selected.

### 4.12. Adhesion to HaCaT Cells

To study the inhibition of *C. auris* adhesion to HaCaT cells monolayers by SphA-L, cells were pre-treated with the concentrations corresponding to ½ MIC and 1 MIC for 2 h at 37 °C and 5% CO_2_. Afterwards, yeast inoculum (2 × 10 ^7^ cells ml^−1^) was added, and each plate was incubated for 2 h at 37 °C and 5% CO_2_. The infected monolayers were rinsed three times with PBS and cells were recovered and diluted in PBS. The number of viable adherent yeast was determined by serial dilution and plating on YPD agar plates.

### 4.13. Caenorhabditis elegans Infection Study

Besides the antifungal efficacy, WT Bristol N2 *C. elegans* nematodes were used for in vivo safety assessments and toxicity evaluations of SphA and SphA-L, which were performed according to previous studies [45]. The nematodes were maintained on nematode growth medium (NGM) plates seeded with *E. coli* OP50 and maintained at 20 °C. Briefly, 40 L4 synchronized nematodes obtained from adult nematodes were washed with M9 buffer (3 g L^−1^ KH_2_PO_4_, 6 g L^−1^ Na_2_HPO_4_, 5 g L^−1^ NaCl, 0.12 g L^−1^ MgSO_4_) and placed into each well of 24-well plates containing M9 buffer (500 μL) with SphA or SphA-L (range: between 5 and 25 μg mL^−1^). The number of surviving worms on each plate was verified 24 h post exposure. Results were expressed as percentages of live worms, counting the dead nematodes under a microscope (40× magnification) compared to the control group, which represented 100% of survival. Infection plates were prepared by inoculating *C. auris* on NGM plates, which were incubated at 37 °C for 24 h to establish a bacterial lawn. Approximately 100 L4-stage or young adult worms were transferred to the infection plates, being placed near the bacterial growth to avoid contamination with *E. coli* from the previous cultures. To evaluate the efficacy of SphA and SphA-L, the infected nematodes were transferred to fresh plates containing *C. auris* (at final concentration of 1 × 10^6^ CFU mL^−1^) with the two compounds at a concentration of 10 μg mL^−1^. Any worms that failed to show movement were recorded as dead and removed to avoid interfering with other nematodes. Additionally, the reproductive output of the infected nematodes was measured by counting the number of offspring per living nematode.

### 4.14. Data Analysis

Data analysis was conducted using GraphPad Prism software (version 8.02 for Windows, GraphPad Software, La Jolla, CA, USA, www.graphpad.com, accessed on 24 October 2024). The results were expressed as means ± standard deviations (SDs) and were obtained from two or three independent experiments. For comparative analysis, one-way or two-way analysis of variance (ANOVA) followed by Tukey’s test was utilized.

## Figures and Tables

**Table 1:** Sizes, expressed as z-average, as measured by DSL and polydispersity index (PDI). Data are expressed as means ± standard deviations (SDs) of three separate experiments for each of two batch formulations, with at least 13 measurements taken for each.

Liposomes	Average Size (d. nm)	PDI	Average Size (d. nm) After 6 Months	% Sph-A
empty liposome	117.0 ± 0.8	0.10 ± 0.01	124.8 ± 0.1	
SphA-L (20 mg mL^−1^)	108.2 ± 0.6	0.10 ± 0.02	128.2 ± 0.1	75%
SphA-L (50 mg mL^−1^)	118.7 ± 1.1	0.13 ± 0.01	124.2 ± 0.2	54%
SphA-L (100 mg mL^−1^)	111.2 ± 0.7	0.12 ± 0.01	126.2 ± 0.3	55%
SphA-L (200 mg mL^−1^)	120.1 ± 1.0	0.10 ± 0.02	125.2 ± 0.1	55%

**Table 2:** Zeta potential measured by DSL. Data are expressed as means ± standard deviations (SDs) of three separate experiments for each of two batch formulations, with at least 13 measurements taken for each.

Liposomes	Zeta Potential (mV)
Liposome-empty	−1.93 ± 0.64
SphA-L (200 μg mL^−1^)	−2.23 ± 0.36

**Table 3:** MIC and MBC of SphA and SphA-L against *C. auris* DSM 21092.

	MIC	MFC	MFC/MIC	
	(μg mL^−1^)		(ratio)	
SphA	35	<100	-	Fungistatic
SphA-L	50	<100	-	Fungistatic

## Data Availability

The original contributions presented in the study are included in the article, further inquiries can be directed to the corresponding author.

## Supplementary Materials

The following supporting information can be downloaded at: https://www.mdpi.com/article/10.3390/molecules29245949/s1, Figure S1. ^1^H NMR spectrum of Sphaeropsidin A (CDCl3, 400 MHz); Figure S2. ESI MS spectrum of Sphaeropsidin A recorded in positive mode; Figure S3. FACS flow cytometry analysis of *C. auris* by using Propidium Iodide staining (PI). Flow cytometry analysis shows the Propidium Iodide staining (DNA content) after *C. auris* synchronization as described in Section 4. The data were analyzed with the 10.6 version of FlowJo Program.
