# Supplementary material for: Sphaeropsidin A Loaded in Liposomes to Reduce Its Cytotoxicity and Preserve Antifungal Activity Against Candida auris
Source: Molecules. 2024 Dec 17;29(24):5949. doi: 10.3390/molecules29245949 (PMC11678014; doi:10.3390/molecules29245949)
Supplement: Supplementary file 1 [file molecules-29-05949-s001.zip › molecules-3324393-supplementary.pdf]

molecules

MDPI

Supplementary Materials

Cytotoxicity and Preserve Antifungal Activity Against *Candida auris*

Annalisa Buonanno [1,†], Maria Michela Salvatore [1,2,†], Antonia Feola [1], Antonietta Siciliano [1], Rosa Bellavita [3], Lorenzo Emiliano Imbò [4,5], Marco Guida [1,6,7], Anna Andolfi [2,6], Rosario Nicoletti [8], Angela Maione [1,*,†], Annarita Falanga [4,5,*,†] and Emilia Galdiero [1]

[1] Department of Biology, University of Naples 'Federico II', Via Cinthia, 80126 Naples, Italy; annalisa.buonanno@unina.it (A.B.); mariamichela.salvatore@unina.it (M.M.S.); antonia.feola@unina.it (A.F.); antonietta.siciliano@unina.it (A.S.); marco.guida@unina.it (M.G.); emilia.galiero@unina.it (E.G.)
[2] Department of Chemical Sciences, University of Naples Federico II, 80126 Naples, Italy; anna.andolfi@unina.it
[3] Department of Pharmacy, School of Medicine, University of Naples 'Federico II', Via Domenico Montesano 49, 80131 Naples, Italy; rosa.bellavita@unina.it
[4] Department of Agricultural Science, University of Naples 'Federico II', Via Università 100, 80055 Portici, Itay; lorenzo.emiliano.imbo@gmail.com
[5] CiRPEB, Research Centre on Bioactive Peptides "Carlo Pedone", University of Naples "Federico II", 80134, Naples, Italy
[6] BAT Center—Interuniversity Center for Studies on Bioinspired Agro-Environmental Technology, University of Naples Federico II, 80055 Portici, Italy
[7] National Biodiversity Future Center (NBFC), 90133 Palermo, Italy
[8] Council for Agricultural Research and Economics, Research Center for Olive, Fruit, and Citrus Crops, 81100 Caserta, Italy; rosario.nicoletti@crea.gov.it
* Correspondence: angela.maione@unina.it (A.M.); annarita.falanga@unina.it (A.F.)
† These authors contributed equally to this work.

Supporting Information List

Figure S1. ^{1}H NMR spectrum of Sphaeropsidin A ($CDCl_3$, 400 MHz).

Figure S2. ESI MS spectrum of Sphaeropsidin A recorded in positive mode.

Figure S3. FACS flow cytometry analysis of *C. auris* by using Propidium Iodide staining (PI). Flow cytometry analysis shows the Propidium Iodide staining (DNA content) after *C. auris* synchronization as described in Material and Methods. The data were analyzed with the 10.6 version of FlowJo Program

 https://doi.org/10.3390/molecules29245949

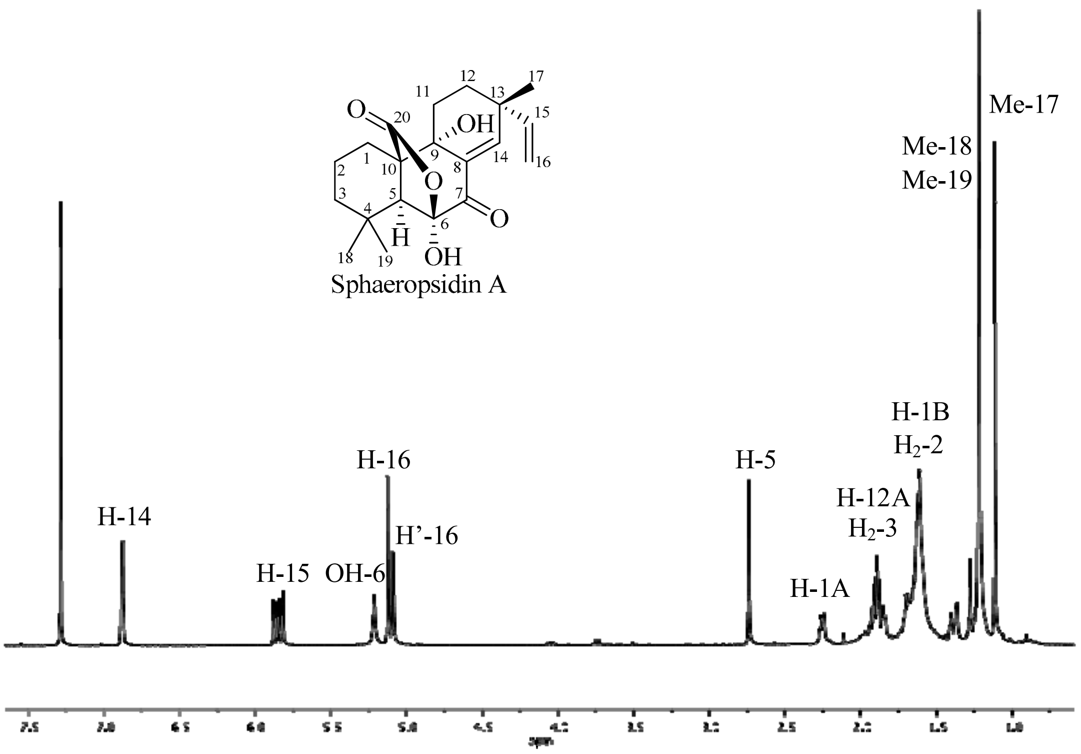

Figure S1. ^{1}H NMR spectrum of Sphaeropsidin A ($CDCl_3$, 400 MHz).

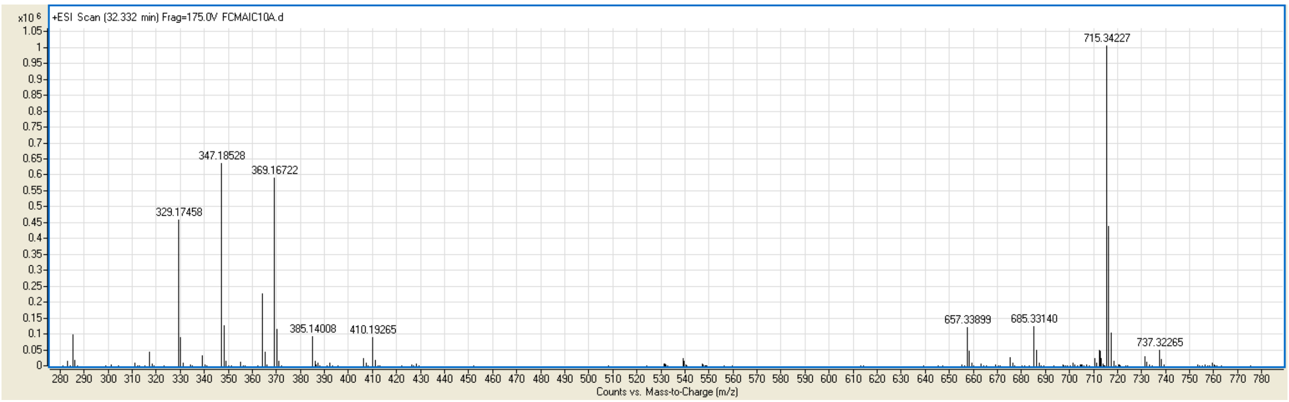

Figure S2. ESI MS spectrum of Sphaeropsidin A recorded in positive mode.

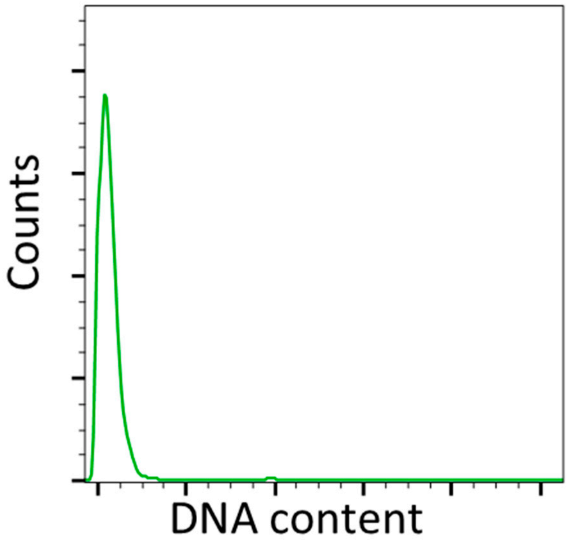

Figure S3. FACS flow cytometry analysis of *C. auris* by using Propidium Iodide staining (PI). Flow cytometry analysis shows the Propidium Iodide staining (DNA content) after *C. auris* synchronization as described in Material and Methods. The data were analyzed with the 10.6 version of FlowJo Program.

Disclaimer/Publisher's Note: The statements, opinions and data contained in all publications are solely those of the individual author(s) and contributor(s) and not of MDPI and/or the editor(s). MDPI and/or the editor(s) disclaim responsibility for any injury to people or property resulting from any ideas, methods, instructions or products referred to in the content.
